# Assessment of hematopoietic failure due to Rpl11 deficiency in a zebrafish model of Diamond-Blackfan anemia by deep sequencing

**DOI:** 10.1186/1471-2164-14-896

**Published:** 2013-12-17

**Authors:** Zhaojun Zhang, Haibo Jia, Qian Zhang, Yang Wan, Yang Zhou, Qiong Jia, Wanguang Zhang, Weiping Yuan, Tao Cheng, Xiaofan Zhu, Xiangdong Fang

**Affiliations:** 1 CAS Key Laboratory of Genome Sciences and Information, Beijing Institute of Genomics, Chinese Academy of Sciences, Beijing 100101, China; 2 Key Laboratory of Molecular Biophysics of Ministry of Education, College of Life Science and Technology Center for Human Genome Research, Huazhong University of Science and Technology, Wuhan, Hubei 430074, China; 3 State Key Laboratory of Experimental Hematology, Institute of Hematology and Blood Disease Hospital, Chinese Academy of Medical Sciences & Peking Union Medical College, Tianjin 300020, China; 4 Hepatic Surgery Center Tongji Hospital, Tongji Medical College, Huazhong University of Science and Technology, Wuhan, Hubei 430074, China

**Keywords:** Zebrafish, Hematopoiesis, Rpl11, RNA-Seq, Transcriptome, DBA

## Abstract

**Background:**

Diamond–Blackfan anemia is a rare congenital red blood cell dysplasia that develops soon after birth. RPL11 mutations account for approximately 4.8% of human DBA cases with defective hematopoietic phenotypes. However, the mechanisms by which RPL11 regulates hematopoiesis in DBA remain elusive. In this study, we analyzed the transcriptome using deep sequencing data from an Rpl11-deficient zebrafish model to identify Rpl11-mediated hematopoietic failure and investigate the underlying mechanisms.

**Results:**

We characterized hematological defects in Rpl11-deficient zebrafish embryos by identifying affected hematological genes, hematopoiesis-associated pathways, and regulatory networks. We found that hemoglobin biosynthetic and hematological defects in Rpl11-deficient zebrafish were related to dysregulation of iron metabolism-related genes, including *tfa*, *tfr1b*, *alas2* and *slc25a37*, which are involved in heme and hemoglobin biosynthesis. In addition, we found reduced expression of the hematopoietic stem cells (HSC) marker *cmyb* and HSC transcription factors *tal1* and *hoxb4a* in Rpl11-deficient zebrafish embryos, indicating that the hematopoietic defects may be related to impaired HSC formation, differentiation, and proliferation. However, Rpl11 deficiency did not affect the development of other blood cell lineages such as granulocytes and myelocytes.

**Conclusion:**

We identified hematopoietic failure of Rpl11-deficient zebrafish embryos using transcriptome deep sequencing and elucidated potential underlying mechanisms. The present analyses demonstrate that Rpl11-deficient zebrafish may serve as a model of DBA and may provide insights into the pathogenesis of mutant RPL11-mediated human DBA disease.

## Background

Ribosomal protein dysfunction causes abnormal ribosomal particle assembly and affects protein synthesis in all cell types. Although RP dysfunction affects multiple tissues and systems, pathological defects in hematopoietic systems, such as aberrant development in Diamond–Blackfan anemia (DBA), are the most frequently observed phenotypes [1-3].

Mutations in RP genes reduce the efficiency of ribosome biogenesis, which is indispensable for immature erythrocytes in early and rapid growth phases [4]. Defective ribosome biosynthesis leads to excessive release of RPs such as RPL5 and RPL11, which may increase p53 activity by activating the RPS MDM2-p53 signaling pathway [5]. Because erythroid progenitor cells are highly sensitive to p53 activation [6], subsequent activation of the p53 pathway in erythroid progenitor cells leads to cell cycle arrest and apoptosis and finally to hematopoietic disorders. Interestingly, these hematopoietic defects were rescued by increased RP expression or reduced p53 activity in zebrafish and in erythroid progenitor cells from DBA patients [7-10]. It was also observed that Rpl22l1 plays essential roles in hematopoiesis, but acts independently of p53 and does not induce apoptosis [11].

DBA is a rare congenital red blood cell dysplasia that develops soon after birth. This disorder is mainly characterized by reduced reticulocyte counts, selective red cell aplasia, and macrocytosis, while a number of other blood cell lineages, such as neutrophils and platelets, are maintained or slightly decreased. Furthermore, DBA patients suffer other developmental defects, such as short stature, cleft lip, hand deformities, kidney and heart hypoplasia, and tumor predisposition [12-14]. Ribosome biogenesis defects have been identified as a major cause of DBA, and mutations of genes encoding RPs have emerged as the main cause of DBA. Indeed, various mutations have been found in coding and noncoding regions of at least 13 RPs, including *RPL5, RPS7, RPL9, RPS10, RPL11, RPS15, RPS17, RPS19, RPS24, RPS26, RPL36, RPS27a*, and *RPL35a*, accounting for about 55% of all DBA cases [15-23]. *RPS19* was the first identified causative gene in DBA patients, and its mutations account for 25% of DBA cases [24].

RPL11 is one of 79 vertebrate RPs, and its gene mutations occur on coding and intronic binding regions of chromosome 1 [17]. DBA patients with mutations in *RPL11* show typical hematopoietic defects, and 67% of these have physical deformities, especially of the thumb [17]. In particular, RPL11 dysfunction results in abnormal erythrocyte development, markedly decreased progenitor cell proliferation, delayed erythroid differentiation, and markedly increased apoptosis [25]. Importantly, these are different from the deficiencies in DBA patients with *RPS19* mutations [25]. Hence, divergent mechanisms underlie hematopoietic defects in DBA patients with different RP mutations. Moreover, RPL11 dysfunction in erythroblasts lead to aberrant erythroblast differentiation, reduced hemoglobin, and unusual cell morphology [26].

Zebrafish are a classic animal model for mechanistic studies of embryonic development, hematopoiesis, and DBA [27,28]. Knockdown of most RP genes causes developmental defects in the brain of zebrafish, indicating that the vast majority of RP functions are associated with nervous system development [29]. Several reports focus on regulation of zebrafish hematopoiesis by the RP proteins Rps7, Rpl11, Rps19, and Rps29 [8-10,30]. Rpl11 dysfunction in zebrafish embryos leads to defective development, hematopoiesis, brain development, and shape dysplasia [8,29,31]. Furthermore, Danilova *et al.* reported that the hematopoietic defects caused by Rpl11 dysfunction were fully reversed by the inhibition of p53 activity in zebrafish mutants [8], indicating that p53 may regulate Rpl11 in zebrafish [25,31]. However, hematopoiesis in zebrafish with dysfunctional Rps19 is not fully recovered by inhibition of p53 [32]. Presumably, these varying phenotypic defects in zebrafish embryos with dysfunctional Rpl11 are regulated by multifactorial interactions of genes, regulatory networks, and signaling pathways.

High-throughput transcriptome sequencing is now widely accepted as a useful tool for investigating human disease mechanisms and gene functions. However, this technology has not been widely used in zebrafish research. Using microarray technology, Danilova *et al*. (2011) studied the impact of Rpl11 deficiency on hematopoiesis of zebrafish embryos. However, because of the limitations of detectable gene transcripts, some important aspects of Rpl11 functions in zebrafish may only be elucidated using high-throughput sequencing.

In this study, we examined genome-wide transcription by high-throughput transcriptome sequencing of Rpl11-deficient zebrafish embryos. In combination with bioinformatic analyses, we studied affected genes, hematopoiesis-related signaling pathways, and molecular regulatory networks in Rpl11-deficient zebrafish embryos. We found that Rpl11 deficiency leads to defects of hemoglobin synthesis and hematopoiesis, which were caused by dysregulation of iron metabolism-related genes and impaired HSC differentiation and proliferation. These analyses provide new insights into the pathogenesis of mutant Rpl11-mediated DBA.

## Results

### Hematopoietic defects in Rpl11-deficient zebrafish embryos

**Figure 1:**
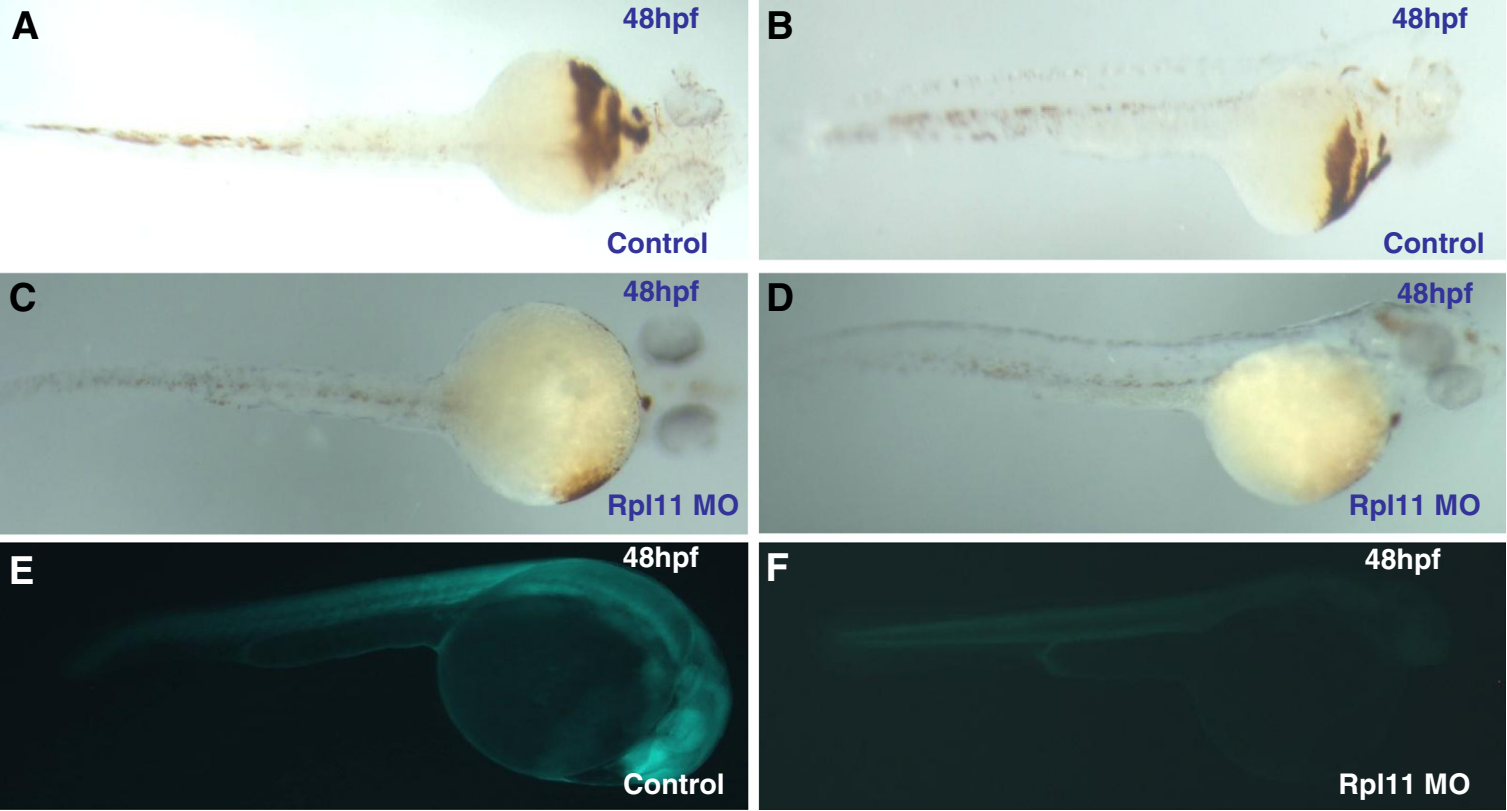
**Benzidine staining of Rpl11-deficient zebrafish embryos and the effectiveness of translational inhibition by Rpl11 MO. (A, B, C, D)**, O-staining shows markedly reduced numbers of hemoglobin-stained blood cells in Rpl11-knockdown embryos; **(E, F)**, The Rpl11–egfp construct was assembled by inserting a partial sequence of Rpl11 cDNA (containing 60 bps from the 5′ UTR) and the N-terminus of egfp into modified pEGFP-N1 (the ATG codon of EGFP was removed). The sequence of Rpl11 MO1 compliments 1–24 bp of Rpl11 cDNA. Embryos co-injected with 25 ng Rpl11–EGFP DNA and 0.5 ng control MO expressed EGFP, and Rpl11–EGFP expression was inhibited by co-injection with 0.5 ng Rpl11 MO; **B, D, E,** and **F** are the lateral view; **A** and **C** are the ventral view.

To investigate the regulation of Rpl11 in hematopoiesis in zebrafish embryos, we initially established an Rpl11-deficient zebrafish model using MO knockdown technology. We observed various phenotypes in this model, including smaller brains, tail deformities, and hematopoietic defects, which were consistent with other studies [8,29,31]. Benzidine staining of Rpl11-deficient zebrafish embryos at 48 hpf showed that globin expression was significantly decreased compared with that in control MO embryos, indicating defects of globin synthesis during late erythroid differentiation (Figure 1A–D). Subsequently, deep sequencing was performed to investigate the impact of Rpl11 on the transcriptome of Rpl11-deficient and control MO embryos at 48 hpf. The effectiveness of translational inhibition by Rpl11 MO was confirmed by examining the green fluorescent fusion protein under a fluorescence microscope (Figure 1E–F).

### Transcriptome changes in Rpl11-deficient zebrafish embryos

To comprehensively reflect the impact of Rpl11 deficiency on the transcriptome of zebrafish embryos, we collected 40–50 Rpl11-deficient and MO control zebrafish embryos at 48 hpf from at least three separate experiments and constructed two mRNA-seq sequencing libraries in parallel. High-throughput sequencing was performed on the Hi-Seq2000 sequencing platform in parallel. The number of sequenced gene transcript reads was 35–40 million. Sequencing data were mapped to the latest Zv9 version of the zebrafish genome. Subsequently, 15–17 million readable gene transcripts were mapped to the zebrafish genome with 41–45% mapping efficiency (Additional file 1: Table S1). On average, more than 10,000 gene transcripts were mapped to known zebrafish genes, accounting for at least 70% of annotated genes. The percentage of detected unique gene transcripts was similar to that detected in previous studies [33,34], indicating that the present sequencing represented the vast majority of zebrafish genes.

All sequenced gene transcript reads were standardized to FPKM [35], and gene expression was presented (Additional file 2: Table S2). The R package DEGseq was used to compare transcriptomes of Rpl11-deficient zebrafish embryos and MO control embryos. In total, we identified 572 significantly differentially expressed genes (DEGs, FC >2, p-value <0.05); 483 were downregulated and 89 were upregulated. We also identified DEGs using screening thresholds set at p-value <0.05 and FC >1.5 (Additional file 3: Table S3). In addition, we found 10 specifically expressed genes (p-value <0.05) that were only expressed in one sample (Additional file 4: Table S4). Among these, four were significantly upregulated in Rpl11-deficient zebrafish embryos, whereas expression of the other six was almost completely inhibited, indicating high dependence of gene expression on Rpl11.

### Gene ontology analysis of Rpl11 in zebrafish embryos

To investigate the biological functions of Rpl11 in zebrafish embryos, we performed GO analyses of genes that were significantly regulated by Rpl11 deficiency using AmiGO software (http://www.geneontology.org/). Since IPA software does not contain the zebrafish gene annotation that must be transformed into human genes before analysis, we used the GO analysis tool in the ZFIN database to get more detailed information of Rpl11 functions in zebrafish. GO analyses showed that upregulated genes were specifically enriched in functions associated with iron metabolism, including iron transport, intracellular iron homeostasis, and iron binding (Additional file 5: Figure S1 and Additional file 6: Table S5). Three zebrafish genes were involved in these iron-related functions, including *zgc:194125, zgc:109934, and zgc:173594* (Additional file 7: Figure S3). However, no detailed gene annotations exist in the latest version (Zv9) of the zebrafish genome. Interestingly, a single human mitochondrial ferritin (FTMT) homolog was found for all three of these zebrafish genes, and its aberrant expression resulted in accumulation of mitochondrial iron, disruption of intracellular iron homeostasis [36], and reduction in iron utilization for heme and hemoglobin synthesis [37]. Downregulated genes were specifically enriched in functions associated with development and differentiation of the nervous system, generation of neurons and brain development, molecular metabolic regulation, developmental regulation of cells and tissues, regulation of gene transcription, regulation of RNA synthesis and metabolism, and other functions (Additional file 5: Figure S2 and Additional file 6: Table S5). Thus, GO analyses demonstrated that Rpl11 participates in the regulation of multiple biological processes in zebrafish embryos, and particularly in hematopoietic iron metabolism-associated pathways.

### Hematological genes affected by Rpl11 deficiency in zebrafish embryos

**Figure 2:**
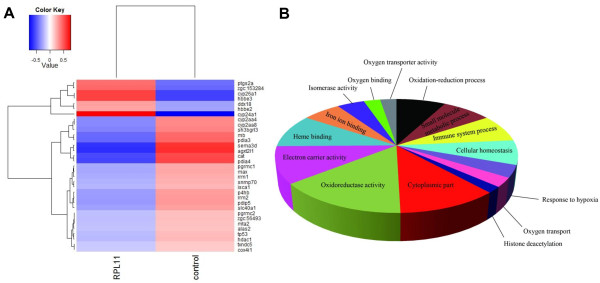
**Analysis of affected hematological genes in Rpl11-deficient zebrafish embryos at 48 hpf. A**, Cluster analysis of affected hematological genes. A total of 33 hematological genes exhibited changed expression in Rpl11-deficient zebrafish embryos at 48 hpf (FC >1.5, p-value <0.05). Hematological genes were clustered in upregulated and downregulated groups, and most affected hematopoietic genes were downregulated in Rpl11-deficient zebrafish embryos at 48 hpf; **B**, Gene ontology (GO) enrichment of affected hematological genes in Rpl11-deficient zebrafish embryos at 48 hpf. Among hematological genes, the largest three fractions represented oxidoreductase activity, cytoplasmic component, and electron carrier activity. Hematological genes with iron metabolism-associated functions such as heme and iron binding were also enriched.

To further investigate hematopoietic failures caused by Rpl11 deficiency in zebrafish, we analyzed affected hematological genes in Rpl11-deficient zebrafish embryos at 48 hpf. To provide a more detailed list of hematological genes affected by Rpl11 deficiency, the screening criteria were set at FC >1.5 and p-value <0.05. In total, 33 differentially expressed hematological genes were identified (Additional file 8: Figure S4), and description of these genes were summarized in Table 1. Heatmap analyses showed that most (24/33) hematological genes were significantly downregulated by Rpl11 deficiency (Figure 2A). Subsequent GO analyses demonstrated that these hematological genes were specifically enriched in diverse hematopoiesis-associated biological processes, including oxidation–reduction processes, heme binding, hypoxic oxygen binding, oxygen transporter activity, iron transport, and oxygen transport (Figure 2B). Hematological genes involved in GO items were summarized in (Additional file 9: Table S6).

**Table 1:** Affected hematological genes in Rpl11-deficient zebrafish embryos at 48 hpf

**Genes**	**Fold change**	**p-Value**	**Description**	**Human homolog**
hbbe3	↑3.12	0	Hemoglobin beta embryonic-3	HBE1
hbbe2	↑1.56	3.84E-23	Hemoglobin beta embryonic-2	HBE1
zgc:153284	↑2.32	6.20E-13	zgc:153284	-
cyp26a1	↑3.13	0.001089	Cytochrome P450, subfamily XXVIA, polypeptide 1	CYP26A1
ddx18	↑1.58	0.003224	DEAD (Asp-Glu-Ala-Asp) box polypeptide 18	DDX18
cyp24a1	↑6.16	0.014658	Cytochrome P450, family 24, subfamily A, polypeptide 1	CYP24A1
ptgs2a	↑2.21	0.023441	Prostaglandin-endoperoxide synthase 2a	-
zgc:56493	↓1.63	2.38E-18	zgc:56493	TXN
cox4i1	↓1.52	6.39E-17	Cytochrome c oxidase subunit IV isoform 1	COX4I1
pdia3	↓2.94	1.45E-13	Protein disulfide isomerase family A, member 3	PDIA3
pdia4	↓3.75	7.23E-10	Protein disulfide isomerase associated 4	PDIA4
hdac1	↓1.68	8.99E-08	Histone deacetylase 1	HDAC1
Cat	↓3.66	2.19E-06	Catalase	CAT
rrm2	↓2.04	1.47E-05	Ribonucleotide reductase M2 polypeptide	-
p4hb	↓2.02	2.57E-05	Procollagen-proline, 2-oxoglutarate 4-dioxygenase, beta polypeptide	P4HB
rrm1	↓1.82	3.28E-05	Ribonucleotide reductase M1 polypeptide	RRM1
cyp2aa8	↓2.29	0.000119	cytochrome P450, family 2, subfamily AA, polypeptide 8	-
tp53	↓1.66	0.000172	Tumor protein p53	TP53
agxt2l1	↓4.63	0.000349	Alanine-glyoxylate aminotransferase 2-like 1	AGXT2L1
pdip5	↓2.06	0.000366	Protein disulfide isomerase-related protein (provisional)	PDIA6
alas2	↓1.60	0.000435	Aminolevulinate, delta-, synthetase 2	ALAS2
Max	↓1.88	0.000645	myc-associated factor X	MAX
isca1	↓1.72	0.000755	Iron-sulfur cluster assembly 1	ISCA1
sh3bgrl3	↓2.39	0.002398	SH3 domain binding glutamic acid-rich protein like 3	SH3BGRL3
pgrmc1	↓1.86	0.003269	Progesterone receptor membrane component 1	PGRMC1
snrnp70	↓1.76	0.006001	Small nuclear ribonucleoprotein 70 (U1)	SNRNP70
cyp2aa4	↓2.27	0.007172	Cytochrome P450, family 2, subfamily AA, polypeptide 4	-
mta2	↓1.62	0.009737	Metastasis associated 1 family, member 2	MTA2
sema3d	↓4.42	0.016997	Semaphorin 3d	SEMA3D
txndc5	↓1.53	0.029683	Thioredoxin domain containing 5	TXNDC5
Mb	↓2.85	0.037113	Myoglobin	MB
slc40a1	↓1.96	0.037939	Solute carrier family 40 (iron-regulated transporter), member 1	SLC40A1
pgrmc2	↓1.63	0.038689	Progesterone receptor membrane component 2	PGRMC2

Differentially expressed hematological genes in Rpl11-deficient zebrafish embryos were identified (Fold change >1.5, p-value <0.05).

### Identification of hematopoiesis-associated pathways disturbed in Rpl11-deficient zebrafish

Key signaling pathways in mammals are highly conserved in zebrafish. To elucidate the roles of Rpl11 in the regulation of hematopoiesis in zebrafish embryos, we used IPA software to identify the signaling pathways that are most affected (p-value <0.01) by Rpl11 deficiency. Separate pathways were constructed with upregulated and downregulated genes (Additional file 10: Table S7). Among the five most affected signaling pathways, we identified three hematopoiesis pathways that were upregulated, including glucocorticoid receptor signaling [38,39], IGF-1 signaling [40-42], and IL-17A signaling in fibroblasts [43], and two hematopoiesis-related pathways that were downregulated, including Wnt/β-catenin signaling [44-48] and Aryl hydrocarbon receptor signaling [49] (Table 2). Among these pathways, Wnt/β-catenin signaling has been characterized as essential for formation, self-renewal, and development of hematopoietic stem cells (HSCs) [48], which are closely associated with hematopoiesis. Taken together, Rpl11 deficiency disturbed hematopoiesis-related signaling pathways in zebrafish embryos, at least partially explaining the ensuing hematopoietic failure.

**Table 2:** Affected hematopoiesis-associated signaling pathways in Rpl11-deficient zebrafish embryos at 48 hpf

**Ingenuity canonical pathways**	**-log (p-value)**	**Up/down regulation**	**Functions**	**Reference**
Wnt/β-catenin signaling	3.81E + 00	Down	HSC formation, self-renewal, and differentiation	[44-48]
Aryl hydrocarbon receptor signaling	3.70E + 00	Down	Maintenance of HSC quiescence	[49]
IGF-1 signaling	4.28E + 00	Up	Regulation of hematopoiesis and proliferation of hematopoietic stem cell progenitor cells	[40-42]
IL-17A signaling in fibroblasts	4.27E + 00	Up	Linking T cell function and hematopoiesis, mediating the hematopoietic response	[43]
Glucocorticoid receptor signaling	3.67E + 00	Up	Stimulation of BFU-E progenitor self-renewal and regulation of stress erythropoiesis	[38,39]

### Identification of regulatory networks affected by Rpl11 deficiency in zebrafish embryos

We used IPA software to determine whether molecular regulatory networks were disturbed by Rpl11 deficiency in zebrafish embryos (Additional file 11: Figure S5A-C, Figure S6A-E, and Additional file 12: Table S8). Analyses showed no specifically enriched hematopoiesis-associated regulatory networks of differentially regulated genes. Potentially, hematopoiesis-associated networks may have been weakened by more important functions of Rpl11 in zebrafish embryos and were therefore not detectable or were obscured by networks regulating hematopoiesis by unknown mechanisms. Through these networks, Rpl11 may play critical regulatory roles in cell development, proliferation, and apoptosis, nervous system development, embryonic development, immunological disease, and tumor pathology. We also identified central network nodes that may directly or indirectly associate with RPL11, including MMP9, AP1, SERPINE1, CTGF, FOS, JUNB, SOCS3, SOX2, TWIST1, CCND1, MMP2, and APP.

### Potential mechanisms through which Rpl11 regulates hematopoiesis in zebrafish embryos

**Figure 3:**
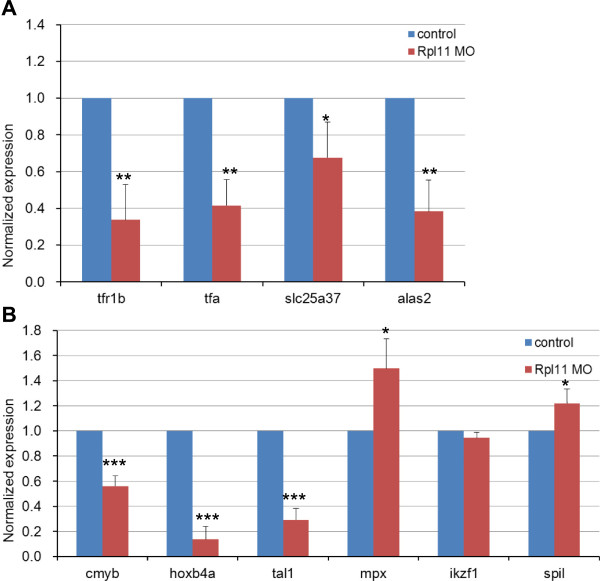
**qPCR analysis of changes in the expression of genes potentially associated with hematological defects in Rpl11-deficient zebrafish embryos. A**, Analysis of changes in expression of genes associated with iron and heme metabolism in Rpl11-deficient zebrafish embryos; **B**, Analysis of changes in expression of molecular blood cell lineage markers. Gene expression was represented as mean ± SD and One-way ANOVA was performed for comparison between MO control and Rpl11-deficient embryos (***P < 0.001, **P < 0.01, * < 0.05, n = 3). Gene expression in MO control samples was normalized to 1.

Benzidine staining of Rpl11-deficient zebrafish embryos showed that hemoglobin biosynthesis was significantly diminished, suggesting defects in hemoglobinization and late erythroid differentiation, which are consistent with the hypochromic anemia typical of iron, heme, or globin deficit in zebrafish embryos [50]. Focusing on the genes associated with erythrocyte iron metabolism, we found that the majority of genes involved in zebrafish iron metabolism were abnormally expressed (Table 3). These included *alas2*, *cp*, *fth1a/b*, *htt*, *aco2*, *slc25a37*, *sfxn1*, *tfa*, and *tfr1b*, suggesting that Rpl11 deficiency in zebrafish might affect multiple aspects of iron metabolism that are closely regulated in highly hemoglobinized erythrocytes. For example, reduced expression of *tfa* and *tfr1b* transcripts indicates damaged iron acquisition by erythrocytes and that of *slc25a37* and *alas2* indicates aberrant iron utilization by erythrocytes (Figure 3A). Moreover, upregulation of three homologs of human FTMT in Rpl11-deficient zebrafish embryos, including *zgc:194125*, *zgc:109934*, and *zgc:173594* (Additional file 7: Figure S3), could lead to imbalances in cytoplasmic and mitochondrial iron metabolism in erythrocytes [37]. At present, it is widely reported that dysfunction of these genes in animal models leads to hematopoiesis-associated diseases such as siderocytic and microcytic anemia (Table 3). Taken together, aberrant expression of these genes may seriously damage iron metabolism, iron supply for heme biosynthesis, and hemoglobin biosynthesis during late erythroid differentiation, finally resulting in defective hematopoiesis in zebrafish embryo.

**Table 3:** Summary of affected iron metabolism-related genes in Rpl11-deficient zebrafish embryos

**Zebrafish gene**	**Fold change**	**Functions**	**Hematopoiesis-associated phenotypes**	**Human homolog**	**Reference**
alas2	↓1.44	First enzyme of heme synthesis	X-linked sideroblastic anemia	ALAS2	[51]
tfa	↓1.89	Fe(III)-carrier in plasma	Severe anemia	TF	[52]
tfr1b	↓2.75	Membrane receptor for Fe(II)-TF	Embryonic lethality and hematopoietic defect	TFRC	[53]
aco2	↓1.3	Bind to IREs; Fe sensors	Microcytic anemia	IRP2	[54]
fth1a	↓1.35	Prevents Fe toxicity during erythropoiesis	Embryonic lethality	FTH1	[55,56]
fth1b	↓1.83				
slc25a37	↓1.77	Iron transport into mitochondria	Embryonic lethal with profound anemia; hypochromic anemia	SLC25A37	[57,58]
cp	↓2.88	Oxidizes exported Fe2+	Hypochromic microcytic anemia	CP	[59]
htt	↓1.27	Transferrin receptor trafficking	Hypochromic anemia	HTT	[60]
sfxn1	↓2.19	Mitochondrial transport and iron metabolism	Siderocytic anemia	SFXN1	[61]
zgc:194125	↑2.73	Iron homeostasis and heme synthesis	Sideroblastic anemia	FTMT	[37,62]
zgc:109934	↑2.36				
zgc:173594	↑4.12				

The arrow up and down respectively stand for up- and down-regulation of genes in Rpl11-deficient zebrafish embryos.

HSCs are capable of self-renewal and have the potential to differentiate into all blood cell lineages, including erythrocytes. Runt-related transcription factor 1 (Runx1) was previously shown to be necessary for formation of HSCs. However, mRNA expression of *runx1* was too low (FPKM <1) to represent its actual expression in zebrafish embryos using RNA-Seq data. Thus, we assessed the proliferation and differentiation of HSCs in Rpl11-deficient zebrafish embryos by observing the expression of the HSC marker *cmyb*. As shown in Figure 3B and Additional file 13: Table S9, we observed a marked decrease in the mRNA expression of *cmyb*, indicating reduction in HSCs in Rpl11-deficient zebrafish embryos. In addition, we observed dramatic reductions in mRNA expression of HSC transcription factors, including *hoxb4a*, which regulates HSC expansion [63], and *tal1*, which controls differentiation and development of HSCs [64]. Given occasional dysplasia of blood cell lineages other than erythrocytes in DBA patients, we determined whether Rpl11 deficiency affected the development and proliferation of granulocytes, lymphocytes, and myelocytes by observing the cellular markers *ikzf1*, *spi1*, and *mpx*, respectively. qPCR analyses showed no changes in expression of these markers in Rpl11-deficient zebrafish embryos (Figure 3B), suggesting that development and proliferation of lymphocytes, myelocytes, and granulocytes were unaffected by Rpl11 deficiency. Taken together, hematopoietic failure in Rpl11-deficient zebrafish may be attributed to defective erythropoiesis in zebrafish embryo HSCs.

## Discussion

### P53 pathway acts independently of Rpl11-deficiency in zebrafish embryos

In previous studies, hematopoiesis was sensitive to p53 activation [6], and p53 pathway was shown to be activated in DBA patients [25,65] and in RP-deficient zebrafish with defective hematopoiesis [9,10,30]. However, we did not observe upregulation of p53 signaling in Rpl11-deficient zebrafish embryos. Potentially slight changes in expression of p53-related genes were obscured by more marked transcriptional consequences of Rpl11 deficiency in zebrafish embryos. However, these observations are consistent with RPL11-independent activation of the p53 pathway in DBA patients [25]. In addition, p53 expression was decreased in tumor cells with dysfunctional RPs [66], suggesting that p53 expression may be sensitive to tumor and tissue environments. Consistent with cells of DBA patients, we speculate that cells of Rpl11-deficient zebrafish embryos may carry characteristics of tumor cells [13,17,67].

**Figure 4:**
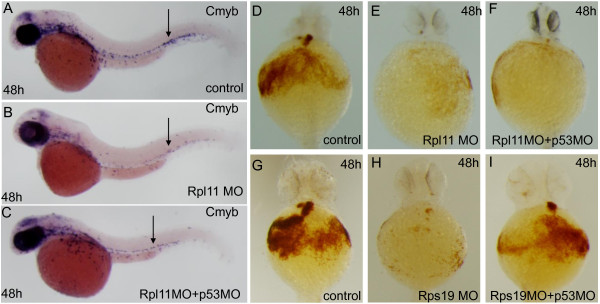
**Rpl11 deficiency is required for hematopoietic defects through p53-independent pathways in zebrafish embryos. A**-**C**, Rpl11 is required for HSC formation through p53-independent pathways. The expression of *cmyb* was significantly decreased in Rpl11 morphants and in Rpl11 and p53 double morphants at 48 hpf compared with that in control embryos. **D**-**I**, Rpl11 deficiency caused defective hematopoietic phenotypes in zebrafish embryos through p53-independent pathways. Hemoglobin staining of embryos individually injected with Rpl11 MO or Rps19 MO and coinjected with p53 MO were performed. **D** and **G** are controls, **E** and **H** are Rpl11 MO or Rps19 knockdown, and **F** and **I** are Rpl11 MO and Rps19 MO coinjected with p53 MO, respectively. Panel **A**–**C**, lateral views; **D**-**I**, ventral views.

It remains unclear whether the entire hematopoietic defective phenotype of DBA is associated with increased expression of p53 or p53-independent mechanisms [25]. Indeed, in a recent report, dysfunctional Rpl22 and Rpl22l1 led to blockage of HSC emergence. Although Rpl22l1 plays essential roles in hematopoiesis, it did not induce apoptosis and acted independently of p53 [11]. In this study, we tested the effects of p53 on phenotypes of Rpl11-deficient zebrafish embryos by observing changes in the expression of the HSC formation marker *cmyb* in *Rpl11* morphants and *Rpl11* and *p53* in double morphants at 48 hpf. In these experiments, decreased *cmyb* expression was not significantly rescued by p53 MO at the base of the Rpl11 MO construct (Figure 4A-C). In addition, we also tested the effects of p53 on defective hematopoietic phenotypes of Rpl11-deficient zebrafish embryos by performing hemoglobin staining of embryos injected with Rpl11 MO and Rps19 MO, both individually and with coinjections of p53 MO. O-staining showed a significant reduction in the number of hemoglobin stained blood cells in individual Rpl11 or Rps19 knockdowns. The Rps19 MO phenotype was partially rescued by coinjection with p53 MO, whereas the Rpl11 MO phenotype was not (Figure 4D-I). Taken together, these results demonstrated that Rpl11 leads to hematopoietic defects in zebrafish embryos through p53-independent pathways.

### Hemoglobin genes’ translation could be inhibited in Rpl11-deficient zebrafish embryos

We observed markedly reduced hemoglobin expression in Rpl11-deficient zebrafish embryos and analyzed globin expression using RT-PCR analysis data (Additional file 14: Figure S7). In total, we identified six globin transcripts *hbaa1*, *hbae1*, *hbae3*, *hbbe1*, *hbbe2*, and *hbbe3* in zebrafish embryos at 48 hpf in RNA-Seq data. Among these, *hbae1*, *hbae3*, *hbbe1*, and *hbbe3* are specifically expressed in embryos, while *hbbe2* and *hbaa1* are specifically expressed in larvae and adults, respectively [68]. Quantitative PCR analyses demonstrated decreased *hbaa1* and *hbbe1* expression, but increased *hbae1*, *hbae3*, *hbbe2*, and *hbbe3* expression to varying degrees (Additional file 14: Figure S7). Given the reduced availability of heme and iron in Rpl11-deficient zebrafish, we deduced that translation of globins might depend on the availability of heme [69]. Because RPL11 deficiency specifically impairs the translation of erythroid genes in murine and human erythroblasts [26], we hypothesized that Rpl11 may specifically inhibit erythroid-specific globin translation in zebrafish embryos.

### Dysfunction of iron- and heme-metabolism related genes in Rpl11-deficient zebrafish

Erythrocyte hemoglobinization involves upregulation and close integration of the heme synthesis and iron supply pathways during hematopoiesis. In Rpl11-deficient zebrafish, the vast majority of iron and heme metabolism-related genes were downregulated, contributing significantly to defective hemoglobinization and hematopoiesis. Hematopoiesis requires large amounts of iron. In serum, most iron is bound to transferrin (Tf; tfa in zebrafish), which directs iron to hemoglobin via transferrin receptor (TfR1; tfr1b in zebrafish)-mediated endocytosis [70,71]. TfR1 (tfr1b) is highly expressed in erythrocytes and is directly associated with hemoglobin synthesis and erythroid progenitor maturation [71,72]. On binding, the Fe(III)–transferrin–TfR1 complex is rapidly internalized, and iron atoms are released and directed to the mitochondria for heme synthesis [70,71]. The mitochondrial iron transporter SLC25A37 (slc25a37 in zebrafish) is also highly expressed in erythrocytes, and its activity has been shown to be rate limiting for heme synthesis in erythrocytes [57,58]. Moreover, deficiency of the *Drosophila* homolog mfrn causes a 90% decrease in heme synthesis, and knockout of Slc25a37 in mice leads to embryonic lethality with profound anemia [58]. Zebrafish with the mutant *slc25a37* show severe hypochromic anemia due to defective mitochondrial iron uptake [57]. ALAS2 is an erythroid-specific rate-limiting enzyme responsible for synthesis of protoporphyrin IX, which is transported out of the mitochondria and installed in globin chains for hemoglobin biosynthesis [73]. Indeed, mutations of *Alas2* in mice caused sideroblastic anemia [51]. Finally, FTMT was not expressed in normal erythroblasts but was highly expressed in patients with sideroblastic anemia [62]. Upregulation of FTMT results in accumulation of mitochondrial iron followed by disruption of intracellular iron homeostasis [36]. Moreover, overexpression of FTMT limits the availability of iron for heme synthesis and impairs mitochondrial iron metabolism [37]. We also observed increased expression of the putative zebrafish FTMT homologs *zgc:194125*, *zgc:109934*, and *zgc:173594* in Rpl11-deficient zebrafish embryos, further indicating hematopoiesis-associated disease exists in Rpl11-deficient zebrafish. Taken together, these observations suggest that Rpl11 participates in zebrafish hematopoiesis as a regulator of iron and heme metabolism.

### Rpl11-deficient zebrafish could serve as a human DBA model

*RPL11* gene mutation leads to disease phenotypes of human DBA, including hematopoietic defects and physical deformities [18]. In zebrafish, Rpl11 deficiency leads to hematopoietic and metabolic defects [8] as well as brain developmental abnormalities [31]. Zebrafish with dysfunctional RPs have been regarded as suitable animal models for DBA studies [30]. Using the Rpl11-deficient zebrafish model and transcriptome deep sequencing, we have added crucial details to the understanding of the biological functions of Rpl11 in zebrafish and have elucidated potential mechanisms by which Rpl11 regulates hematopoiesis. Future studies will determine which pathways and regulatory mechanisms are common to both human DBAs and this zebrafish model. Zebrafish may serve as model systems for human diseases, and hence, our findings provide an important resource and shed new insights for the study of human DBA diseases.

## Conclusion

**Figure 5:**
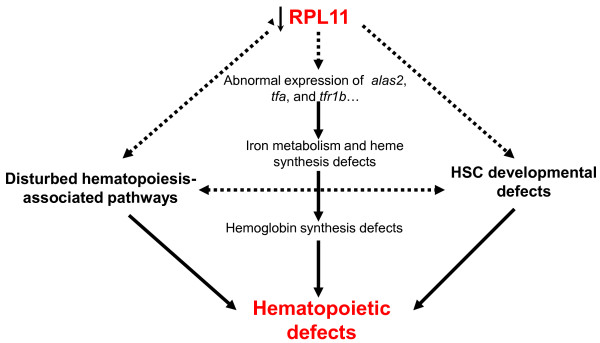
**Potential molecular mechanisms by which Rpl11 deficiency leads to hematopoietic defects in zebrafish embryos.** In this study, we deduced three potential mechanisms by which Rpl11 deficiency leads to hematopoietic defects in zebrafish embryos. First, Rpl11 deficiency led to abnormal expression of a set of iron metabolism- and heme synthesis-related genes. Second, Rpl11 deficiency disturbed proliferation and development of HSCs. Finally, Rpl11 deficiency disturbed several hematopoiesis-associated pathways. Solid lines represent mechanisms that have been confirmed in this or other studies, and dashed lines represent mechanisms that require further validation.

In this study, we established an Rpl11-deficient zebrafish model that showed defective hemoglobinization and hematopoiesis. To elucidate the mechanisms of hematopoietic failure, we performed mRNA transcriptome deep sequencing of Rpl11-deficient and control MO zebrafish embryos and systematically characterized Rpl11-mediated disturbances in hematological gene expression, hematopoiesis-related signaling pathways, and regulatory networks in zebrafish. We also elucidated potential mechanisms by which Rpl11 regulates hematopoiesis through genes associated with iron metabolism and proliferation and development of HSCs in zebrafish embryos or through hematopoiesis-associated pathways with unknown mechanisms (Figure 5). The present data provide new insights into the pathogenesis of RPL11-mediated DBA.

## Methods

### Zebrafish embryo maintenance, Rpl11 morpholino (MO) microinjection, and hemoglobin staining

Wild-type zebrafish (Danio rerio; AB type) were maintained under standard library conditions and zebrafish embryo stages (hours post fertilization; hpf) were determined as described previously [74,75]. All studies of zebrafish were approved by the Animal Care and Use Committee of Huazhong University of Science and Technology. Rpl11 Morphlino (MO; 5-CTTCTTCTCGCTCTGGTCCGCCATG-3) and control MO (5-CTTATTCGCGCTATGGTCGGCAATG-3) were obtained from Gene-Tools, LLC. The Rps19 MO, p53 MO, and control MO were previously described [76]. Zebrafish embryos at the one-cell stage were injected with MOs using a microinjector (WPI SYS-PV830). Based on the literature and our initial injection trials, 0.5-ng Rpl11 MO and control MO were chosen as the optimal concentration. The effectiveness of translational inhibition by this Rpl11 MO was tested *in vivo* using a Rpl11–egfp green fluorescent fusion protein. We found that at a dose of 0.5 ng/embryo, most GFP expression was knocked down. Hemoglobin in zebrafish embryos was analyzed using o-dianisidine (Sigma) as described [77]. All images were collected using an Olympus microscope with a digital camera (OLYMPUS IX71) and were imported into Adobe Photoshop CS2 9.0.2 for orientation and figure preparation.

### Total RNA isolation, library preparation, and sequencing

Immediately after harvesting, 40–50 pooled embryos at 48 hpf from at least three experiment were snap-frozen in liquid nitrogen and stored at −80°C. Total RNA was extracted from pooled embryos using TRIzol reagent (Invitrogen) according to the manufacturer’s instructions. RNA concentrations were determined using NanoDrop 2000 (Thermo Scientific). The integrity of RNA samples was determined using 1.2% agarose gel electrophoresis, followed by removal of the residual genomic DNA with RNase-free DNase I (Ambion). Libraries of mRNA were constructed using the Illumina mRNA-Seq library preparation kit according to the manufacturer’s instructions. Concentration and size distribution of the libraries were determined on an Agilent Bioanalyzer DNA 2000 chip (Agilent Technologies), followed by sequencing on an Illumina Hiseq 2000 Genome Analyser platform in pair-end mode by 100-bp lengths. A total of 35–40 million reads were collected for further analysis.

### RNA-Seq data analysis

Reads were processed and aligned to the UCSC zebrafish reference genome (build Zv9/danRer7, Jul. 2010) using TopHat (version 1.3.3) [78]. TopHat incorporates the Bowtie v0.12.7 algorithm to perform alignments. In brief, TopHat initially removes a portion of reads based on the quality of information accompanying each read and maps qualified reads to the reference genome. The reference index was built using Bowtie with a fasta file for the entire genome of zebrafish, which was downloaded from UCSC (http://genome.ucsc.edu/). Parameters were set by default, but the number of threads to align reads was set at 6. TopHat aligned read files were then entered into Cufflinks (version 1.2.1) software for further analyses, including transcript assembly, abundance estimation, and differential expression and regulation testing in RNA-Seq samples [79]. To calculate gene expression intensity, read counts were normalized to the number of fragments per kilobase of transcript per million mapped reads (FPKM) according to the gene length and total mapped reads [78]. Confidence intervals for estimates of FPKM were calculated using a Bayesian inference method [80]. The R package DEGseq was used to identify DEGs from RNA-Seq data. Genes with FPKM less than 1 were removed from analyses. DEGs were characterized according to the criterion of fold change (FC) >2.0 and p-value <0.05.

### RT-PCR

Real-time RT-PCR was performed to verify the expression of genes detected in mRNA-seq experiments involving Rpl11-deficient and MO control zebrafish embryos. Reverse transcription was performed using the cDNA Reverse Transcription Kit (Fermentas) with oligo d(T)s. RT-PCR was performed using SYBR Green PCR Master Mix (Fermentas) with a CFX-96 Real-time PCR system according to the manufacturer’s instructions (Bio-Rad) (Additional file 15: Table S10). PCR products were analyzed using agarose gel electrophoresis to identify bands of expected sizes. Gene expression was calculated according to the real-time PCR manual of Bio-Rad and normalized to the expression of β-actin. Data were represented as mean ± SD and One-way ANOVA was performed for comparison between MO control and Rpl11-deficient embryos.

### IPA analysis

Gene interaction networks and signaling pathways were generated using Ingenuity Pathways Analysis (IPA) software (http://www.ingenuity.com/) from Ingenuity® Systems. This software was used to analyze data from a variety of experimental platforms and to provide accurate biological insights into interactions between genes, proteins, chemicals, pathways, cellular phenotypes, and disease processes. Differentially expressed genes were processed using DEGseq and screened according to the criteria FC >2 and p-value <0.05. These genes were submitted to IPA for biological function, canonical pathway, and interaction network analyses. Since the current edition of IPA cannot accept and identify zebrafish gene IDs, we matched these differentially expressed zebrafish genes with their human homologs using the homolog database HomoloGene, which was downloaded from NCBI. After conversion to human homolog genes, these homologs were submitted to IPA, sets of genes that were enriched for a particular function or pathway were identified, and enrichment ratios were calculated. To determine the significance of enrichment in a particular function, IPA calculates the significance value based on the measure of involvement of the gene in the input data set with its respective molecular functions and signaling pathways [81]. The significance of networks was calculated using Fisher’s exact test, and p-values were executed using negative logarithmic transformation.

### Screening of differentially expressed hematological genes

Zebrafish gene sets associated with hematological systems were identified by searching with the keywords “erythroid,” “hematopoietic,” “globin,” and “hema” using the AmiGO software of the Gene Ontology (GO) database (http://www.geneontology.org/). Differentially expressed genes (FC >2.0, p-value <0.05) detected by DEGseq were mapped to this hematological gene set, which indicated the differentially expressed hematological genes in our data set. Mapping and drawing of the scatter diagrams were accomplished using R scripts.

### Cluster analysis

Expression intensities of differentially expressed hematological genes were used in cluster analyses. Gene expression intensity was normalized using the equation $x_{i}^{'}=\frac{x_{i}-\bar{x}}{\bar{x}}$, where *x*_*i*_ is the FPKM value and $\bar{x}$ is the mean FPKM value of a gene calculated from two samples. Average linkage hierarchical clustering was performed in this study using Pearson distance as the measure between genes and samples. Computation and visualization were achieved using the heatmap plus package in R.

### Data access

Supporting data sets are available in the Gene Expression Omnibus, with the accession number GSE45699. The following link has been created to allow review of record GSE51326 while it remains in private status: http://www.ncbi.nlm.nih.gov/geo/query/acc.cgi?token=mzcbuyyipbixhun&acc=GSE51326.

## Abbreviations

DBA: Diamond–Blackfan anemia; Rpl11: Ribosomal protein L11; HSC: Hematopoietic stem cells; FPKM: The number of fragments per kilobase of transcript per million mapped reads; DEGs: Differentially expressed genes; RPs: Ribosomal proteins.

## Competing interests

The authors declare that they have no competing interests.

## Authors' contributions

ZZ was involved in mRNA-Seq library preparation, carried out bioinformatics studies and data organization, and drafted the manuscript. HJ established Rpl11-deficient zebrafish model, carried out zebrafish related experiments, and participated in data organization. QZ participated in the sequence alignment and bioinformatics analysis. YW, YZ, QJ and WZ participated in zebrafish related experiments. WY and TC participated in its overall design and instruction, and revised it critically for important intellectual content. XF and XZ conceived of the study, participated in coordination, and gave final approval of the version to be published. All authors read and approved the final manuscript.

## Supplementary Material

Additional file 1: Table S1 RNA-seq reads mapping analysis. Click here for file

Additional file 2: Table S2 All gene transcripts detected in Rpl11-deficient and MO control zebrafish embryos using RNA-seq. Click here for file

Additional file 3: Table S3 All DEGs in Rpl11-deficient zebrafish embryos at 48 hpf. This table includes up- and downregulated genes in Rpl11-deficient zebrafish embryos (fold change>1.5 or 2, p-value<0.05). Click here for file

Additional file 4: Table S4 Specifically expressed genes in MO control and Rpl11-deficient zebrafish embryos. Click here for file

Additional file 5 GO enrichment of up- and downregulated genes in Rpl11-deficient zebrafish embryos at 48 hpf. GO enrichment includes three analytical aspects: biological process, molecular function, and cellular component. DEGs in Rpl11-deficient zebrafish embryos at 48 hpf (FC >2, p-value <0.05) were included in this analysis. Blue labeled functions are associated with DEGs in Rpl11-deficient zebrafish embryos. This figure was generated using AmiGO software (http://www.geneontology.org/). Figure S1, GO enrichment of upregulated genes in Rpl11-deficient zebrafish embryos at 48 hpf. Figure S2, GO enrichment of downregulated genes in Rpl11-deficient zebrafish embryos at 48 hpf. Click here for file

Additional file 6: Table S5 Genes involved in GO enrichment of up- and downregulated DEGs in Rpl11-deficient zebrafish embryos. Click here for file

Additional file 7: Figure S3 qPCR analyses of human FTMT homologs in Rpl11-deficient zebrafish embryos at 48 hpf. These genes were *zgc:194125*, *zgc:173594*, and *zgc:109934*, which were specifically enriched in iron metabolism-associated functions such as iron transport and cellular iron homeostasis in GO analyses, and were upregualted in Rpl11-deficient zebrafish embryos (Mean ± SD, one-way ANOVA, **P < 0.01, *<0.05, n = 3). Gene expression in MO control samples was normalized to 1. Click here for file

Additional file 8: Figure S4 Scatter plot of DEGs in Rpl11-deficient zebrafish embryos at 48 hpf. DEGs affected by Rpl11 deficiency in zebrafish embryos at 48 hpf are shown (FC >1.5, p-value <0.05), and affected hematological genes (highlighted red) were further analyzed. Click here for file

Additional file 9: Table S6 Affected hematological genes enriched in GO items. Click here for file

Additional file 10: Table S7 Disturbed signaling pathways in Rpl11-deficient zebrafish embryos. This table includes up- and downregulated signaling pathways in Rpl11-deficient zebrafish embryos at 48 hpf. Click here for file

Additional file 11 Networks affected by Rpl11 deficiency in zebrafish embryos at 48 hpf. Networks were generated using online IPA software, and highly enriched networks were selected (Score > 20). Genes labeled red and green were up- and downregulated, respectively. Figure S5 (A-C), Upregulated networks affected by Rpl11 deficiency in zebrafish embryos at 48 hpf. A, Networks of cellular function and maintenance, small molecule biochemistry, and carbohydrate metabolism. B, Networks of cellular development, cellular growth and proliferation, and connective tissue development and function. C, Networks of cell death and survival, connective tissue disorders, and immunological disease. Figure S6 (A-E), Downregulated networks affected by Rpl11 deficiency in zebrafish embryos at 48 hpf. A, Networks of developmental disorders, skeletal and muscular disorders, and digestive system development and function. B, Networks of cellular development, visual system development and function, nervous system development and function, cellular growth and proliferation, connective tissue development and function, neurological disease, and tissue morphology. C, Networks of cell death and survival, cardiac pulmonary embolism, and cardiovascular disease. D, Networks of cancer, cell cycle, and tissue morphology. E, Networks of cell cycle, cell morphology, and cell-to-cell signaling and interaction. Click here for file

Additional file 12: Table S8 Genes involved in regulatory interacting networks disturbed in Rpl11-deficiet zebrafish embryos. Click here for file

Additional file 13: Table S9 Description of blood cell lineage markers. Description of blood cell lineage markers, including *cmyb*, *hoxb4a*, *tal1*, *mpx*, *spi1*, and *ikzf1*, were shown. Blood cell lineages, including hematopoietic stem cells, granulocytes, myelocytes, and lymphoid progenitors were detected in this study. Fold change represents FPKM in Rpl11-deficient zebrafish embryos compared to that of MO control. Click here for file

Additional file 14: Figure S7 RT-PCR analyses of changes in the expression of globin genes in zebrafish embryos after Rpl11 knockdown. A, RT-PCR analysis of changes in expression of globin genes in zebrafish embryos after Rpl11 knockdown (Mean ± SD, one-way ANOVA, **P < 0.01, *<0.05, n = 3). Gene expression in MO control samples was normalized to 1; B, Description of globin genes detected in deep sequencing samples. Click here for file

Additional file 15: Table S10 RT-PCR primers used in this study. Click here for file
