# Supplementary figures and images for: Assessment of hematopoietic failure due to Rpl11 deficiency in a zebrafish model of Diamond-Blackfan anemia by deep sequencing
Source: BMC Genomics. 2013 Dec 17;14:896. doi: 10.1186/1471-2164-14-896 (PMC3890587; doi:10.1186/1471-2164-14-896)

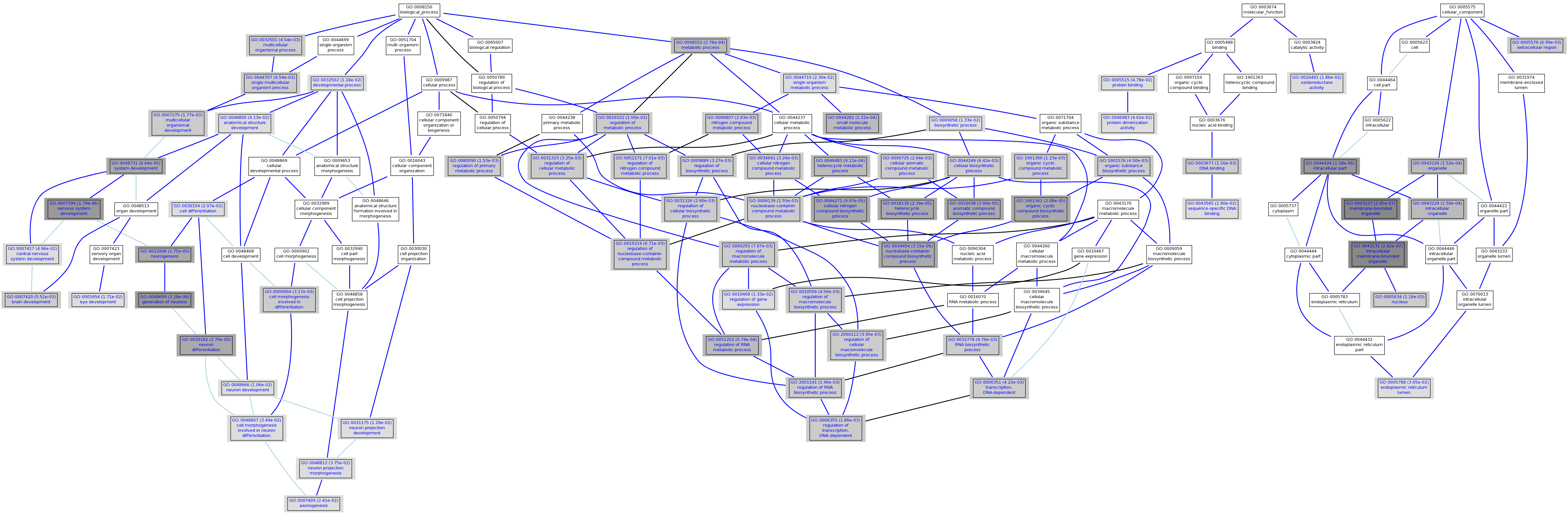

Supplement: Additional file 5 — GO enrichment of up- and downregulated genes in Rpl11-deficient zebrafish embryos at 48 hpf. GO enrichment includes three analytical aspects: biological process, molecular function, and cellular component. DEGs in Rpl11-deficient zebrafish embryos at 48 hpf (FC >2, p-value <0.05) were included in this analysis. Blue labeled functions are associated with DEGs in Rpl11-deficient zebrafish embryos. This figure was generated using AmiGO software (http://www.geneontology.org/). Figure S1, GO enrichment of upregulated genes in Rpl11-deficient zebrafish embryos at 48 hpf. Figure S2, GO enrichment of downregulated genes in Rpl11-deficient zebrafish embryos at 48 hpf. [file 1471-2164-14-896-S5.zip › 5470805769940489_add5.png]

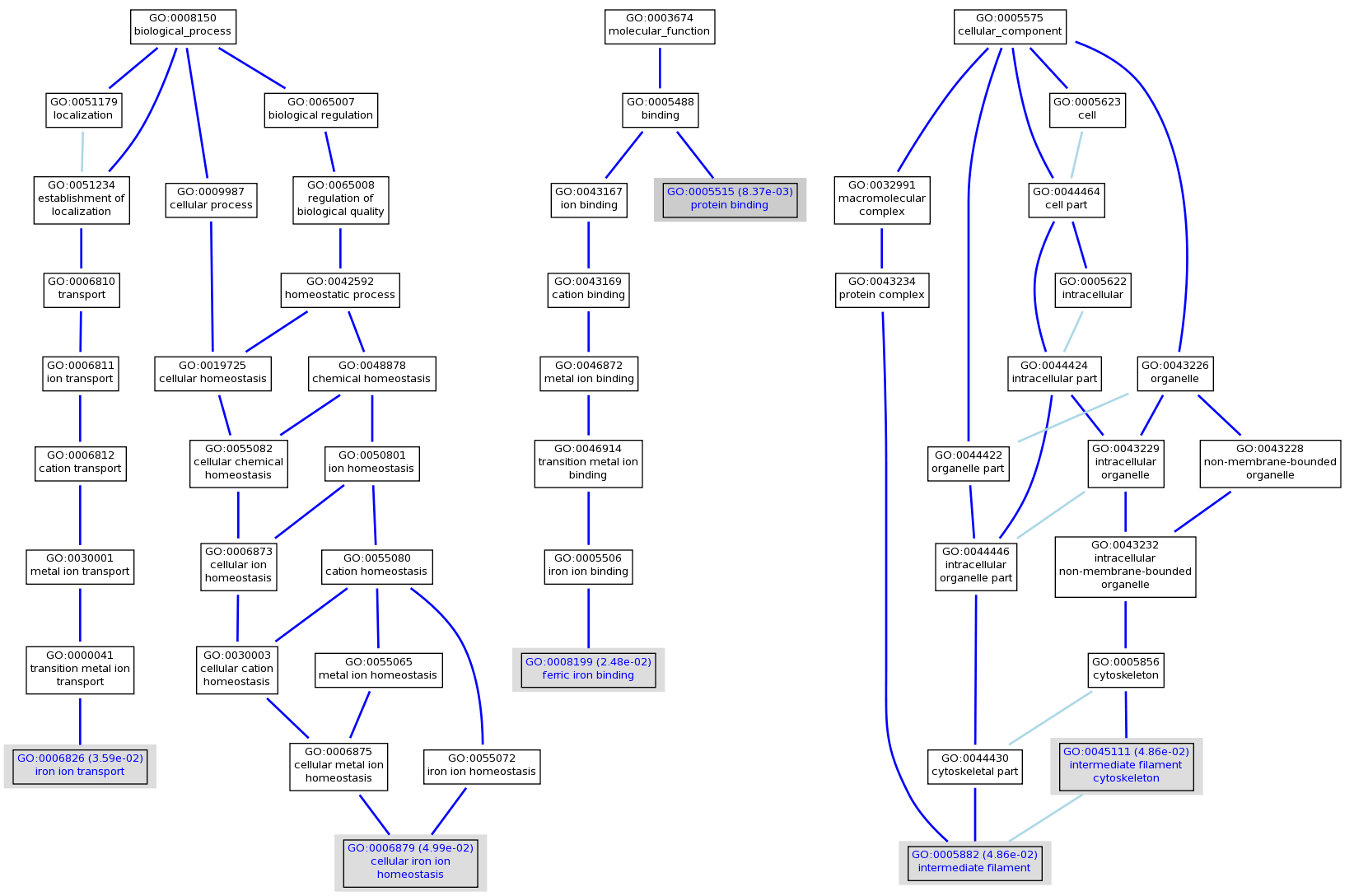

Supplement: Additional file 5 — GO enrichment of up- and downregulated genes in Rpl11-deficient zebrafish embryos at 48 hpf. GO enrichment includes three analytical aspects: biological process, molecular function, and cellular component. DEGs in Rpl11-deficient zebrafish embryos at 48 hpf (FC >2, p-value <0.05) were included in this analysis. Blue labeled functions are associated with DEGs in Rpl11-deficient zebrafish embryos. This figure was generated using AmiGO software (http://www.geneontology.org/). Figure S1, GO enrichment of upregulated genes in Rpl11-deficient zebrafish embryos at 48 hpf. Figure S2, GO enrichment of downregulated genes in Rpl11-deficient zebrafish embryos at 48 hpf. [file 1471-2164-14-896-S5.zip › 5470805769940489_add6.png]

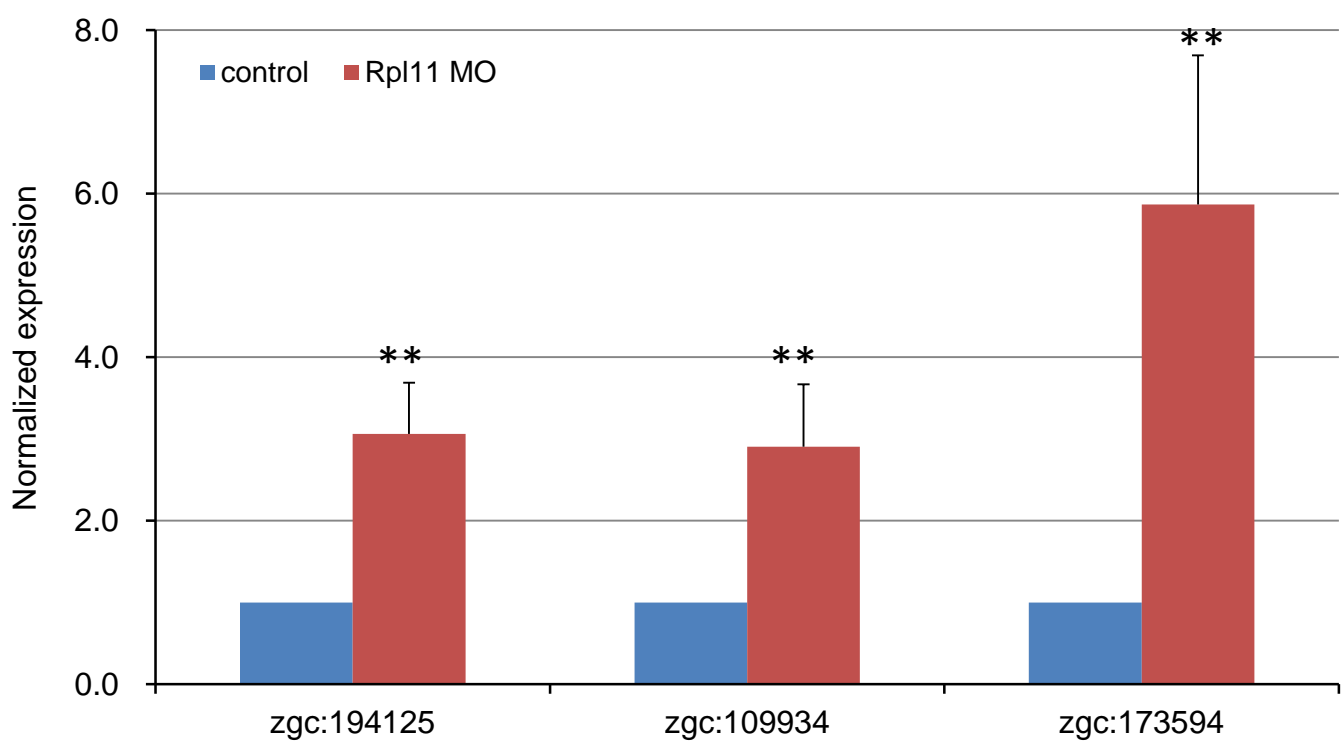

Supplement: Additional file 7: Figure S3 — qPCR analyses of human FTMT homologs in Rpl11-deficient zebrafish embryos at 48 hpf. These genes were zgc:194125, zgc:173594, and zgc:109934, which were specifically enriched in iron metabolism-associated functions such as iron transport and cellular iron homeostasis in GO analyses, and were upregualted in Rpl11-deficient zebrafish embryos (Mean ± SD, one-way ANOVA, **P < 0.01, *<0.05, n = 3). Gene expression in MO control samples was normalized to 1. [file 1471-2164-14-896-S7.pdf]

RPL11 vs. control

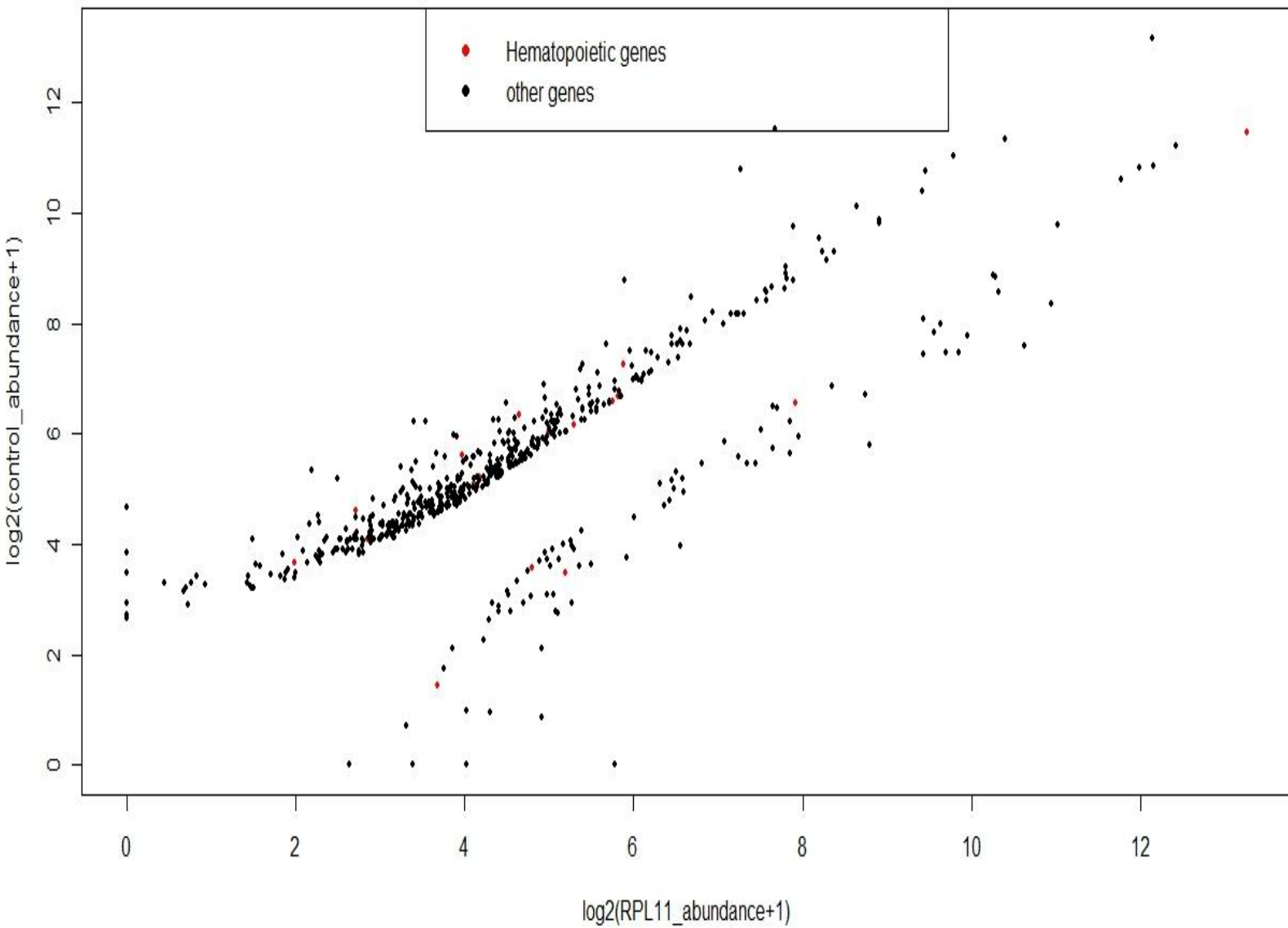

Supplement: Additional file 8: Figure S4 — Scatter plot of DEGs in Rpl11-deficient zebrafish embryos at 48 hpf. DEGs affected by Rpl11 deficiency in zebrafish embryos at 48 hpf are shown (FC >1.5, p-value <0.05), and affected hematological genes (highlighted red) were further analyzed. [file 1471-2164-14-896-S8.pdf]

A

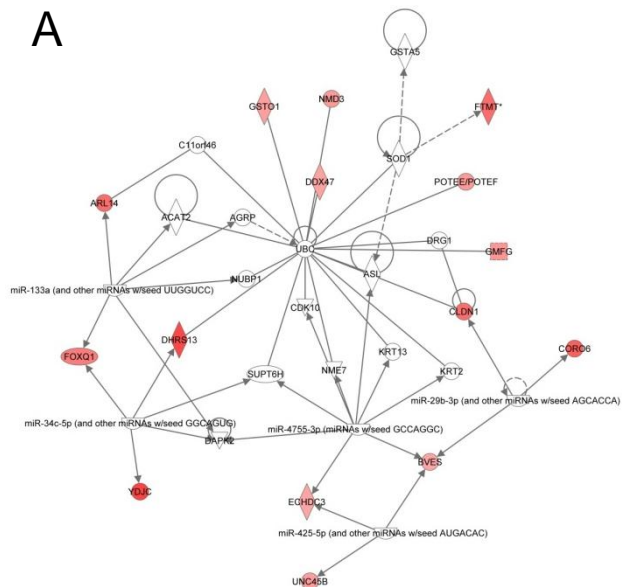

B

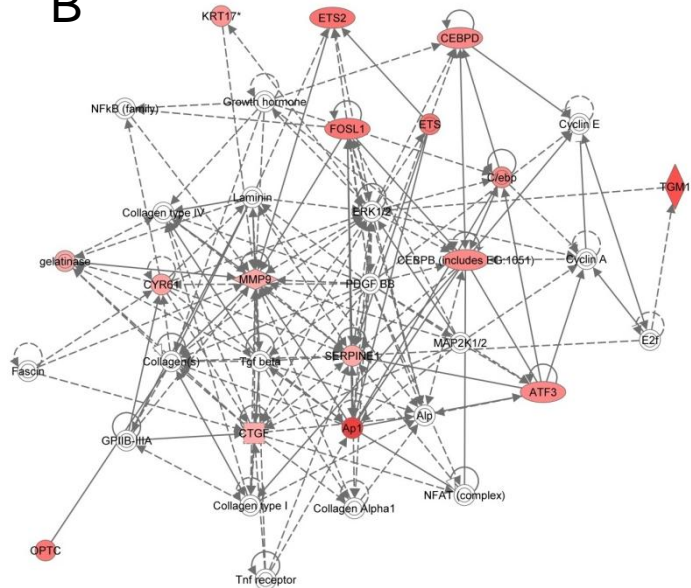

C

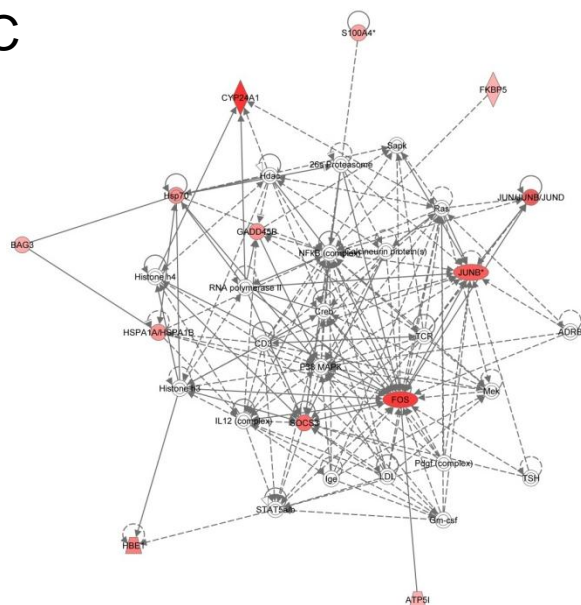

Supplement: Additional file 11 — Networks affected by Rpl11 deficiency in zebrafish embryos at 48 hpf. Networks were generated using online IPA software, and highly enriched networks were selected (Score > 20). Genes labeled red and green were up- and downregulated, respectively. Figure S5 (A-C), Upregulated networks affected by Rpl11 deficiency in zebrafish embryos at 48 hpf. A, Networks of cellular function and maintenance, small molecule biochemistry, and carbohydrate metabolism. B, Networks of cellular development, cellular growth and proliferation, and connective tissue development and function. C, Networks of cell death and survival, connective tissue disorders, and immunological disease. Figure S6 (A-E), Downregulated networks affected by Rpl11 deficiency in zebrafish embryos at 48 hpf. A, Networks of developmental disorders, skeletal and muscular disorders, and digestive system development and function. B, Networks of cellular development, visual system development and function, nervous system development and function, cellular growth and proliferation, connective tissue development and function, neurological disease, and tissue morphology. C, Networks of cell death and survival, cardiac pulmonary embolism, and cardiovascular disease. D, Networks of cancer, cell cycle, and tissue morphology. E, Networks of cell cycle, cell morphology, and cell-to-cell signaling and interaction. [file 1471-2164-14-896-S11.zip › 5470805769940489_add12.pdf]
