# Supplementary material for: Assessment of hematopoietic failure due to Rpl11 deficiency in a zebrafish model of Diamond-Blackfan anemia by deep sequencing
Source: BMC Genomics. 2013 Dec 17;14:896. doi: 10.1186/1471-2164-14-896 (PMC3890587; doi:10.1186/1471-2164-14-896)
Supplement: Additional file 11 — Networks affected by Rpl11 deficiency in zebrafish embryos at 48 hpf. Networks were generated using online IPA software, and highly enriched networks were selected (Score > 20). Genes labeled red and green were up- and downregulated, respectively. Figure S5 (A-C), Upregulated networks affected by Rpl11 deficiency in zebrafish embryos at 48 hpf. A, Networks of cellular function and maintenance, small molecule biochemistry, and carbohydrate metabolism. B, Networks of cellular development, cellular growth and proliferation, and connective tissue development and function. C, Networks of cell death and survival, connective tissue disorders, and immunological disease. Figure S6 (A-E), Downregulated networks affected by Rpl11 deficiency in zebrafish embryos at 48 hpf. A, Networks of developmental disorders, skeletal and muscular disorders, and digestive system development and function. B, Networks of cellular development, visual system development and function, nervous system development and function, cellular growth and proliferation, connective tissue development and function, neurological disease, and tissue morphology. C, Networks of cell death and survival, cardiac pulmonary embolism, and cardiovascular disease. D, Networks of cancer, cell cycle, and tissue morphology. E, Networks of cell cycle, cell morphology, and cell-to-cell signaling and interaction. [file 1471-2164-14-896-S11.zip › 5470805769940489_add13.pdf]

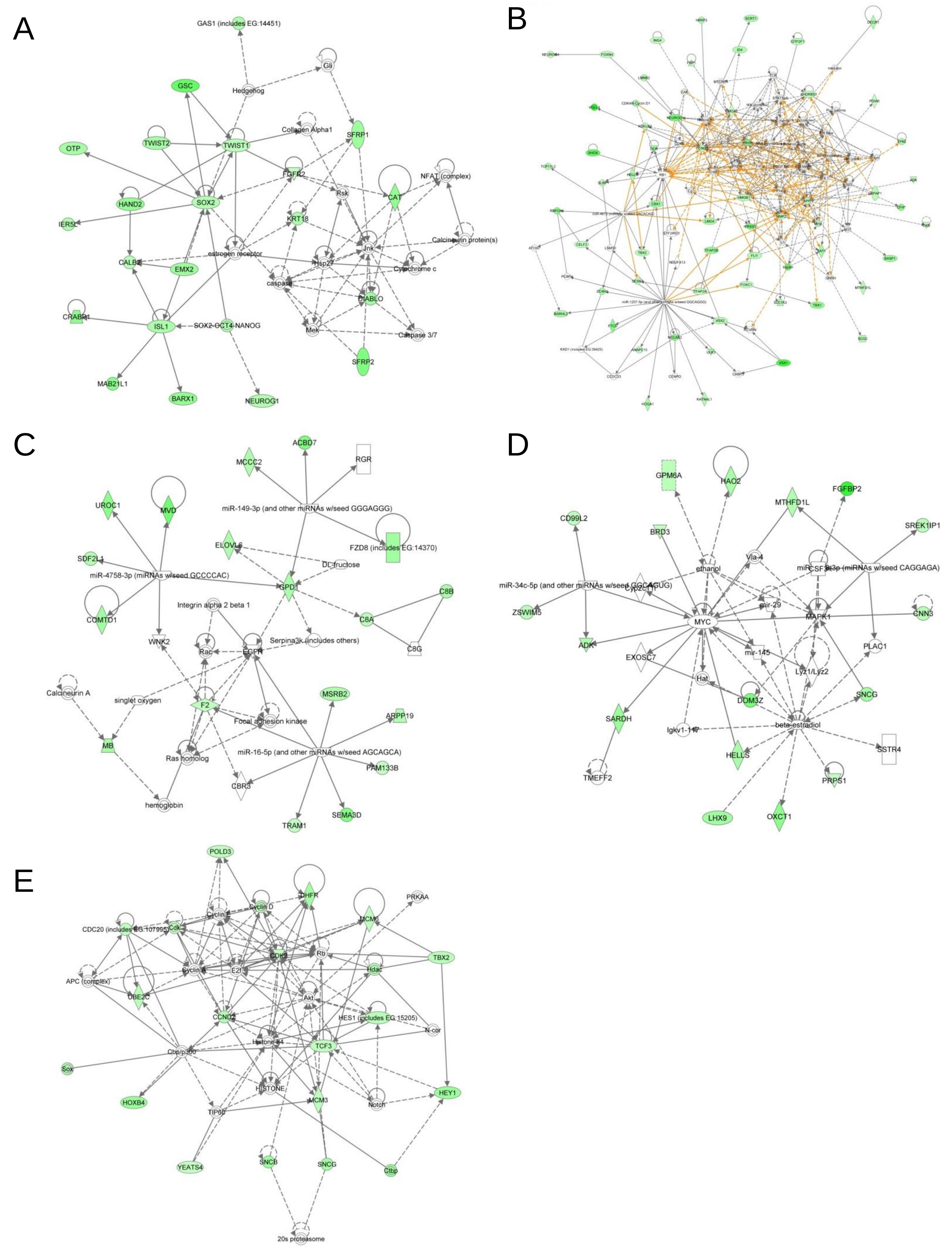

A
GAS1 (includes EG:14451)
Gli
GSC
Hedgehog
Collagen Alpha1
SFRP1
OTP
TWIST2
TWIST1
FGFR2
NFAT (complex)
HAND2
SOX2
Rsk
CAT
IER5L
KRT18
Calcineurin protein(s)
estrogen receptor
Jnk
CALB2
EMX2
Hsp27
Cytochrome c
caspase
DIABLO
CRABP1
ISL1
SOX2-OCT4-NANOG
Mek
Caspase 3/7
SFRP2
MAB21L1
BARX1
NEUROG1
B
C
ACBD7
MCCC2
RGR
UROC1
MVD
miR-149-3p (and other miRNAs w/seed GGGAGGG)
ELOVL6
FZD8 (includes EG:14370)
SDF2L1
DL-fructose
miR-4758-3p (miRNAs w/seed GCCCCAC)
GPD1
C8B
Integrin alpha 2 beta 1
COMTD1
C8A
WNK2
Rac
EGFR
Serpina3k (includes others)
C8G
Calcineurin A
MSRB2
singlet oxygen
F2
ARPP19
Focal adhesion kinase
MB
miR-16-5p (and other miRNAs w/seed AGCAGCA)
Ras homolog
FAM133B
CBR3
hemoglobin
SEMA3D
TRAM1
D
GPM6A
HAO2
FGFBP2
MTHFD1L
CD99L2
BRD3
SREK1IP1
ethanol
Vla-4
miR-34c-5p (and other miRNAs w/seed GGCAGUG)
Cyp2c11
ZSWIM5
mir-29
CNN3
MYC
MAPK1
ADK
PLAC1
EXOSC7
mir-145
Lyz1/Lyz2
Hat
SNCG
DOM3Z
SARDH
beta-estradiol
Igkv1-117
SSTR4
HELLS
TMEFF2
PRPS1
LHX9
OXCT1
E
POLD3
DHFR
PRKAA
Cyclin D
Cyclin E
MCM6
CDC20 (includes EG:107995)
Cdk
CDK2
Rb
TBX2
Cyclin A
E2f
Hdac
APC (complex)
UBE2C
Akt
CCND2
HES1 (includes EG:15205)
N-cor
Histone h4
TCF3
Cbp/p300
Sox
HISTONE
HEY1
HOXB4
MCM3
Notch
TIP60
YEATS4
SNCB
SNCG
Ctbp
20s proteasome
