# Supplementary material for: Assessment of hematopoietic failure due to Rpl11 deficiency in a zebrafish model of Diamond-Blackfan anemia by deep sequencing
Source: BMC Genomics. 2013 Dec 17;14:896. doi: 10.1186/1471-2164-14-896 (PMC3890587; doi:10.1186/1471-2164-14-896)
Supplement: Additional file 14: Figure S7 — RT-PCR analyses of changes in the expression of globin genes in zebrafish embryos after Rpl11 knockdown. A, RT-PCR analysis of changes in expression of globin genes in zebrafish embryos after Rpl11 knockdown (Mean ± SD, one-way ANOVA, **P < 0.01, *<0.05, n = 3). Gene expression in MO control samples was normalized to 1; B, Description of globin genes detected in deep sequencing samples. [file 1471-2164-14-896-S14.pdf]

A

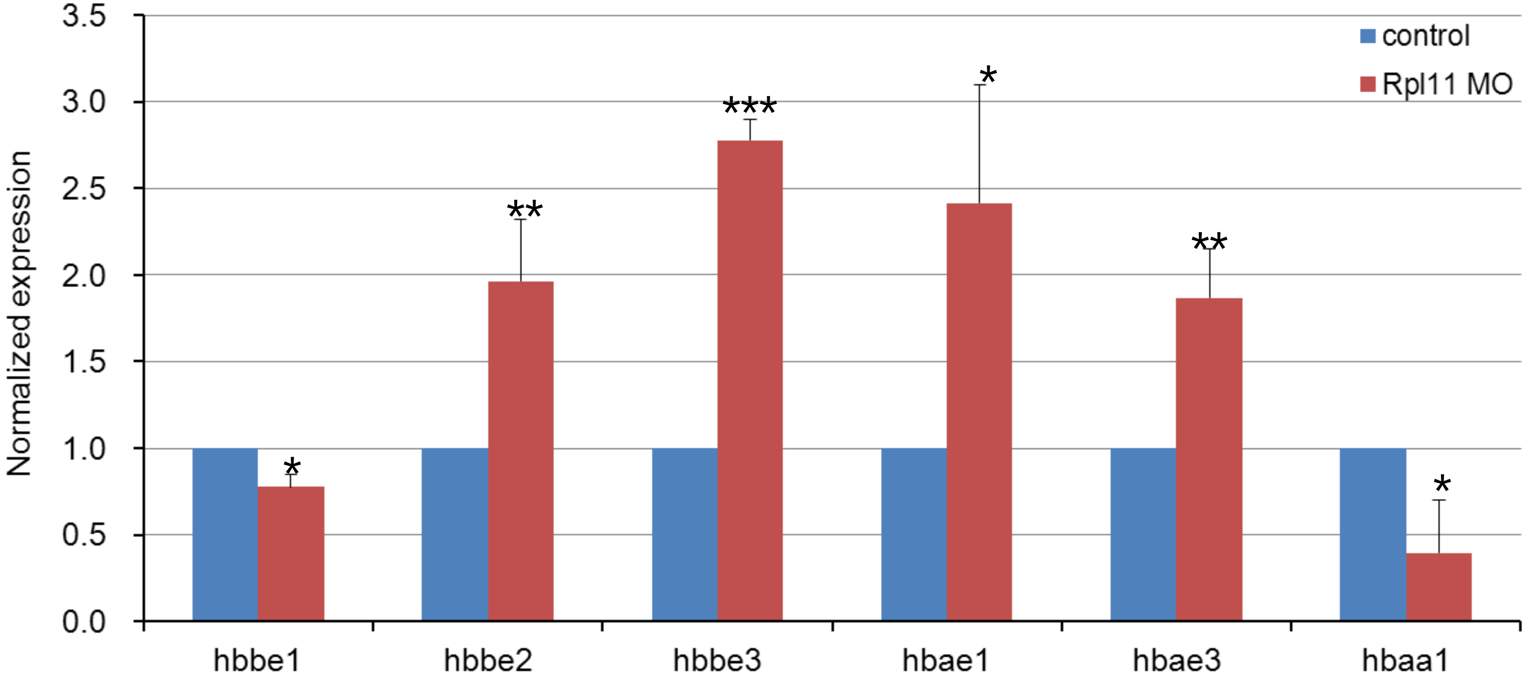

B

	Control_FPKM	RPL11_FPKM	Description
hbaa1	8.78825	6.31559	hemoglobin alpha adult-1
hbae1	4802.32	5644.51	hemoglobin alpha embryonic-1
hbae3	8647.15	11365.1	hemoglobin alpha embryonic-3
hbbe1	3327.64	2509.56	hemoglobin beta embryonic-1.1
hbbe2	766.195	1327.88	hemoglobin beta embryonic-2
hbbe3	2814.22	9779.64	hemoglobin beta embryonic-3
